# Exploring Architectural Details Through a Wearable Egocentric Vision Device

**DOI:** 10.3390/s16020237

**Published:** 2016-02-17

**Authors:** Stefano Alletto, Davide Abati, Giuseppe Serra, Rita Cucchiara

**Affiliations:** Department of Engineering, Università degli Studi di Modena e Reggio Emilia, Via Vivarelli 10, Modena 41125, Italy; stefano.alletto@unimore.it (S.A.); 82189@studenti.unimore.it (D.A.); rita.cucchiara@unimore.it (R.C.)

**Keywords:** computer vision, egocentric vision, smart guides, enhanced tourist experience

## Abstract

Augmented user experiences in the cultural heritage domain are in increasing demand by the new digital native tourists of 21st century. In this paper, we propose a novel solution that aims at assisting the visitor during an outdoor tour of a cultural site using the unique first person perspective of wearable cameras. In particular, the approach exploits computer vision techniques to retrieve the details by proposing a robust descriptor based on the covariance of local features. Using a lightweight wearable board, the solution can localize the user with respect to the 3D point cloud of the historical landmark and provide him with information about the details at which he is currently looking. Experimental results validate the method both in terms of accuracy and computational effort. Furthermore, user evaluation based on real-world experiments shows that the proposal is deemed effective in enriching a cultural experience.

## 1. Introduction

City tourism is growing faster and is deemed more resilient than global tourism, being a significant driving factor for economic growth and entertainment [1]. Historical places, museums and cultural heritage sites are called upon to fulfill this role and must deal with the tourists of the 21th Century, who are beginning to be digital natives expecting multimedia interfaces and augmented experiences. Initial efforts in this sense have been done, e.g., designing immersive environments using touch interfaces and personalized multimedia content [2,3]. For example, in the “SmartMuseum” project [4], visitors can acquire information about the artworks displayed and build a custom visiting path using Personal Digital Assistants (PDAs) and Radio Frequency Identification (RFID). Similarly, RFID technologies are used in [5,6], where the user can interact with tags near the artworks using his mobile device acquiring multimedia contents. Nonetheless, these approaches are still limited to constrained indoor environment and require significant setup efforts. Furthermore, technologies based on RFID infrastructure are poorly scalable and hard to modify and maintain, and are not suitable for outdoor applications.

Consumers are becoming more familiar with wearable cameras and sensors, enabling a wide range of applications among which are augmented cultural experiences. In computer vision, research on the matter has been flourishing for the past years under the name of egocentric vision (or ego-vision). Adopting the very unique first person perspective, it deals with the tasks of self-gestures analysis, social relationships or video summarization [7,8,9,10]. This allows for a new paradigm in algorithm design where the human is in the center and applications are tailored to its needs and expectations. In particular, having a camera that follows the user in a tour of a cultural heritage site and a wearable device that in real-time understands what he is looking at, could be the enabling technology for augmented visits and experiences.

In this paper, we propose to adopt this first person perspective and design a wearable system that follows the tourist in his visit and retrieves architectural details of historical buildings providing information. The main idea is that, in an unconstrained outdoor tour, the user may not be able to autonomously identify all the relevant details in the site he is visiting, so having a smart embedded guide that sees what he sees and can provide precise and contextual information when asked to could result in a more enjoyable and complete experience. In particular, the system we design is composed by a wearable board, a glass-mounted camera, a wireless audio output device and the visitor’s smartphone. Using the latter as interface, the user can ask the system to acquire a frame from the wearable camera, process it, and retrieve all the details present in the scene. The user can then see a visual representation of the output on the smartphone screen, where tapping each detail provides audio information regarding the selected detail.

This work makes two main contributions with respect to its preliminary version proposed in [11]. In particular, we aim at two main improvements: increasing the accuracy of the results providing a more precise retrieval of the details in the scene, and increasing the system responsiveness in order to provide a more natural interface. The former is done by introducing an explicit feature map that, approximating a non-linear kernel, can better capture complex patterns in the data jointly increasing the discriminative capabilities of our descriptor and its scalability. To increase responsiveness, we reduce processing times adopting a new wearable board, namely the Nvidia Jetson TK1, and by parallelizing our algorithms to better exploit this new architecture. While presenting a similar form factor and being in the same price tier of the Odroid XU board used in [11], it allows us to reduce the required time to process a query by nearly five times, resulting in a much more natural interaction with the user. Following these contributions, we perform quantitative evaluation of the actual benefits of the new wearable board comparing the performance of the two different devices. Finally, we provide extended and enriched user experiments, involving a wider sample of people in our use case evaluation.

## 2. Related Work

Visual Landmark Recognition. Since the scope of our work is to provide the user with information about what he is interested in, it becomes crucial to recognize the landmarks he is looking at. Visual object recognition has always been one of the main research topics in computer vision, and in this field significant efforts have been done in proposing solutions for the problem of buildings or visual landmark recognition from query images. For this purpose, large scale datasets have often been used and algorithms can be roughly divided in two main categories [12]: the ones dealing with recognition performance and accuracy [13,14,15,16,17,18], and the ones aiming at real-time processing and reduced computational efforts [19,20,21,22,23,24].

In [13], authors study the effectiveness of image matching for the purpose of building recognition, in particular when performed under significant viewpoint changes. Their pipeline considers the extraction of affine invariant vertical segments and the computation of local descriptors based on both geometrical and color information. To find a matching score between such descriptors, they evaluate the superior performance of Mahalanobis distance used along with euclidean distance between segments. In this work, the setting is constrained to a limited number of possible viewpoints, e.g., images acquired by an automated wheelchair; in contrast, in our egocentric scenario, there are no limits to the possible movements of the camera since it is tied to the user’s head and moves accordingly. Rather different is the proposal by Thrin *et al.* [16]. Addressing the problem of recognizing multiple buildings in a large dataset, they choose to rely on a global image descriptor, rather than focusing on image matching. Their method starts with a facet segmentation step, based on the extraction of line segments and vanishing points information. A descriptor is then associated to each facade by merging a list of Scale Invariant Feature Transform (SIFT) local features [25] with its color histogram, and used for a nearest-neighbor based matching between a query image and its closest model at a later stage. In this work, the authors perform a coarse grained recognition aiming at discriminating one building from another by representing its facade, while in our setting we need to be able to identify different views of different parts of the same building. Aiming at this fine grained recognition, SIFT descriptors are not sufficient due to their inability to correctly discriminate recurrent patterns on a cultural heritage building (see Section 6). Prior to the work by Thrin *et al.* [16] is the proposal by Philbin *et al.* [19], that takes advantage of both the efficiency of global descriptors and the accuracy of local descriptor matching, re-addressing the problem as a ranking one. The algorithm exploits the standard Bag of Words (BoW) model, along with a large codebook, to represent each image as a sparse vector of occurrences of visual words. According to this vector space model, given a query image, the dataset is explored, and a ranked list of relevant results is retrieved according to the cosine similarity. Finally, image matching is performed between the query and the top results, in order to filter out false positives. The use of a large BoW codebook allows the authors to effectively deal with “web-scale” datasets containing millions of images but requires complex optimization and quantization steps to be computationally feasible. On the other hand, in our scenario, the amount of images involved in the creation of the 3D model is limited to few hundreds, thus rendering a similar approach overly complex for the requirements of our application.

The recent work by Weyand *et al.* [26] tries to address some of the open issues in the field, analyzing and testing the full pipeline of a typical visual landmark recognition system from Internet photo collections. Among the issues tackled, the authors discuss about the existence of different types of buildings and their different behaviors in recognition, as well as efficient in-memory image representation and the reliability of user provided semantic information and tagging. About this last theme, they remark the existence of an odd trade-off between the landmark recognition complexity and the information coherence. By clustering images into categories, they show how landmarks belonging to large clusters are easier to classify but usually suffer from noisy information extracted from social channels. On the other hand, reducing the cardinality of the set results in such information becoming more precise, but at the cost of an increased recognition complexity. Once again, the work presents related techniques but in a different scenario, featuring a scale significantly different from the one dealt with in our work.

Architectural detail recognition. The aforementioned techniques, while capable of achieving significant accuracy, aim at a coarse grained recognition: they are in fact able to identify entire buildings but cannot retrieve information on a finer grain such as architectural details. Despite the fact that this task could be approached exploiting semantic information and metadata coupled with images, few works have dealt with this using computer vision techniques. In particular, Weyand and Liebe [27] propose the Hierarchical Iconoid Shift algorithm, based on the analysis of the geometrical structure of a scene given a dataset of images depicting it. Using each image as a seed, they solve a medoid shift problem increasing the kernel bandwidth at every convergence, finding visual modes at different scales (iconoids) using a bottom-up approach. As output of the method, a dendrogram representing the scene structure is built. Differently from our method, where the detail recognition is performed on the 3D model and thus exploits the geodesic constraints of the building itself, the authors cluster 2D images in a hierarchical structure. Differently, the method presented by Mikulik *et al.* [28] follows a top-down methodology: the algorithm is similar to a BoW-based image retrieval system, but in the distance measure between descriptors, a kernel weights the similarity according to the scale change of each visual word, rewarding scale-up (*i.e.*, zoom-in) and penalizing scale-down (zoom-out). While obtaining significant results in the identification of details, the method is computationally demanding and can require up to 38 s on a 2.6 GHz core for a query, thus not being suitable for a real-time embedded application.

**Figure 1:**
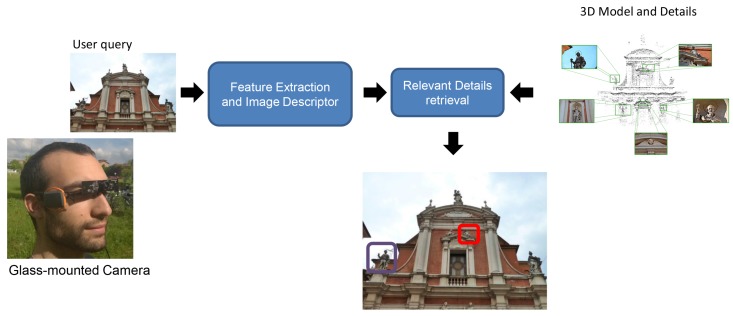
Overall pipeline of the proposed system.

**Figure 2:**
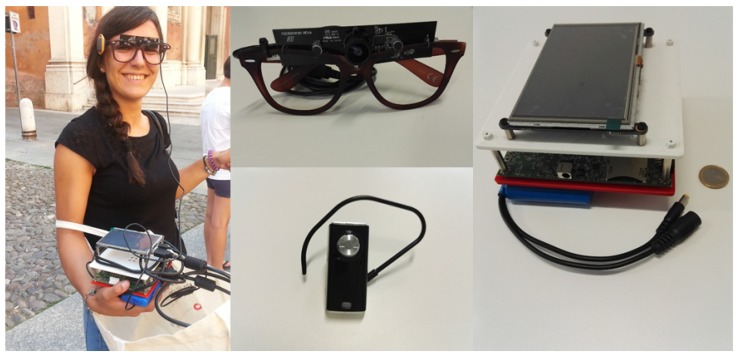
One of the participants in the user evaluation equipped with the hardware provided. Glass-mounted wide-lens camera, Nvidia Jetson TK1 wearable board and bluetooth earphone.

The system built to enable the application features a collection of commercial wearable devices, aiming at low costs and high performance. A schematization of the overall solution is proposed in Figure 1. In particular, the system embeds a glass-mounted wearable camera connected to a Nvidia Jetson TK1 board, a GPS module and a network communication unit. The Nvidia Jetson TK1 board features a 2.32 GHz ARM quad-core Cortex-A15 CPU with Cortex-A15 battery-saving shadow-core and a NVIDIA Kepler “GK20a” GPU with 192 SM3.2 Compute Unified Device Architecture (CUDA) cores (upto 326 GFLOPS). Furthermore, it embeds a high-quality hardware accelerated still-image and video capture path, with dual next-generation Image Signal Processors, which is particularly suitable for the image processing algorithms required by this application. The board is powered by a 3000 mAh battery cell in order to be easily weared in outdoor scenarios. Furthermore, the glass-mounted camera features wide-angle lens, allowing for the capture of a field of view similar to the human capabilities. Figure 2 displays an example of the adopted hardware setup.

## 3. The Proposed Architecture

The wearable board contains a set of 3D models of cultural heritage sites built using structure from motion techniques from unconstrained images (see below, Section 5). Coarse geolocation is obtained using GPS and exploited to select the correct 3D model of the site the user is visiting. This 3D model consists of a 3D point cloud, on which relevant architectural details are highlighted through manual annotation, and the collection of images $C=\{I_{1},\cdots I_{n}\}$ used in its construction. For each of these images, its 2D–3D matches with the point-cloud are stored to facilitate the correspondence search at runtime.

When a new frame is acquired by the application, it is analyzed to retrieve the architectural details contained in it. In particular, SIFT local descriptors are computed and exploited in the construction of the Projected Covariance Descriptor (*pCov*, see Section 4). This descriptor is used in the construction of a ranked list of similar images comparing the global representation of the input query and of the images used in the 3D model building. Euclidean distance is adopted as scoring metric in the comparison of the global descriptors.

After this initial retrieval phase, we propose to refine its results by introducing a spatial verification step that involves the first K top ranked images (we experimentally fix K = 5). The spatial verification process is based on the computation of geometrical transformations between the query image and each top ranked candidate. The match is then scored based on the reprojection of local features that results from the geometrical transformation previously estimated. Following [19], such transformation is estimated though a Random Sample Consensus (RANSAC) loop capable of efficiently dealing with the presence of outliers in the matches. Since the query frames can be acquired by different viewpoints or different zooming conditions, we constrain the RANSAC algorithm to generate affine transformation hypothesis, effectively dealing with rotation and translation warps. Once the method identifies the correct matches for the query, its 2D–3D correspondences with the point cloud are determined using the precomputed matches. These are used to estimate the absolute camera pose by solving the Perspective-n-Point (PnP) problem [29,30]. In order to be able to adopt a wide range of wearable cameras with unknown intrinsic parameters, instead of using the standard three-points solution that indeed requires camera calibration, we adopt the recent approach by Kukelova *et al.* [31] where the PnP problem and the intrinsic parameters estimation are jointly addressed.

Currently, an Android application allows the user to see his captured image with the interesting architectural details highlighted. As future work, we want to extend this application also to run on a head-mounted display that will enable the visitor to obtain a more natural browsing of the contents.

## 4. Projected Covariance Descriptor

Given a set of local SIFT features $F=\{f_{1}\cdots f_{N}\}$ densely extracted from an image *I*, we represent them with a covariance matrix C:

(1) $$C=\frac{1}{N-1}\sum_{i=1}^{N}\left(f_{i}-m\right){\left(f_{i}-m\right)}^{T}$$

where m is the mean vector of the set *F*. Although the covariance matrices encode very rich information (the variance of the features and their correlations), they do not lie in a vector space. In fact, the space of the covariance can be formulated as a differentiable manifold in which Euclidean distance between image descriptors can not be computed. Therefore, in order to use this descriptive representation, we need to define a suitable transformation. Following [32], we exploit a projection from the Riemannian manifold to an Euclidean tangent space, called Log-Euclidean metric. The basic idea of the Log-Euclidean metric is to construct a function map between the Riemannian manifold and the vector space of the symmetric matrices (of which the covariance matrices is a particular case).

First the covariance matrix is projected from Riemannian manifold to an Euclidean tangent space through a specific tangency matrix T. Later, the projected vector is mapped in a orthonormal coordinate system. Matrices (points in the Riemannian manifold) will be denoted by bold uppercase letters, while vectors (points in the Euclidean space) by bold lowercase ones. The projection of C on the hyperplane tangent to T becomes:

(2) $$\Phi ={\mathrm{vec}}_{I}\left(\log\left(T^{-\frac{1}{2}}CT^{-\frac{1}{2}}\right)\right)$$

where log is the matrix logarithm operator and I is the identity matrix, while the vector operator on the tanget space at identity of a symmetric matrix Y is defined as:

(3) $${\mathrm{vec}}_{I}\left(Y\right)=\left[y_{1,1}\;\sqrt{2}y_{1,2}\;\sqrt{2}y_{1,3}\ldots y_{2,2}\;\sqrt{2}y_{2,3}\ldots y_{d,d}\right]$$

By computing the sectional curvature of the Riemmanian manifold, the natural generalization of the classical Gaussian curvature for surfaces, it is possible to show that this space is almost flat [33]. This means that wherever the projection point T is located, the neighborhood distance between the points on the manifold remains unchanged. In addition, from a computational point of view, the best choice for T is the identity matrix, which simply translates the mapping into applying the ${\mathrm{vec}}_{I}$ operator to the standard matrix logarithm. This also frees us from the problem of optimizing the projection point for the specific data under consideration, leading to a generally applicable descriptor. Since the projected covariance is a symmetric matrix of d×d values, the image descriptor *pCov-SIFT* is a $(d^{2}+d)/2$-dimensional feature vector.

### Explicit Feature Map

Non-linear kernels have been successfully applied in vision applications to compare image features, since they can deal with complex patterns in the data [34]. Although they can achieve very good performance in term of accuracy, their major drawback is scalability. In fact, they generally require high computation times and they cannot directly be used in an algorithm that performs fast approximate nearest neighbor searches in high dimensional linear spaces such as the randomized kd-tree forest Fast Library for Approximate Nearest Neighbor (FLANN) [35].

To this end we proposed to use an explicit feature map that linearly approximates the non-liner kernel Chi-Square $\chi^{2}$. In other words, we define a transformation map that projects the feature vector, of an image Φ, in a high dimensional linear space which approximates the $\chi^{2}$ Kernel space and can be directly used as input of FLANN. More formally, the $\chi^{2}$ Kernel is an homogeneous Kernel and is defined as:

(4) $$K\left(x,y\right)=\sum_{i}k\left(x_{i},y_{i}\right)=\sum_{i}\frac{2x_{i}y_{i}}{x_{i}+y_{i}}$$

Based on the specific characteristics of an homogeneous kernel, *k* can represented as:

(5) $$k\left(x,y\right)=\int_{-\infty }^{+\infty }{\left[\Psi (x)\right]}_{\lambda }^{*}{\left[\Psi (y)\right]}_{\lambda }d\lambda$$

where Ψ(x) is the infinite dimensional feature map. Following [36], the Ψ(x) feature map can be approximated by a finite number of samples obtain an efficient representation. The resulting vector $\hat{\Psi }\left(x\right)$ is defined as:

(6) $$\hat{\Psi }{\left(x\right)}_{j}=\left\{\begin{array}{cc} \text{sign}\left(x\right)\sqrt{\left|x|L\kappa (0\right)} & \;\text{if}\;j=0\\ \text{sign}\left(x\right)\sqrt{2\left|x\right|L\kappa \left(\frac{j+1}{2}L\right)}\cos\left(\frac{j+1}{2}L\log\left|x\right|\right) & \;\text{if}\;j>0\;\text{odd}\\ \text{sign}\left(x\right)\sqrt{2\left|x\right|\kappa \left(\frac{j}{2}L\right)}\sin\left(\frac{j}{2}L\log\left|x\right|\right) & \;\text{if}\;j>0\;\text{even} \end{array}\right.$$

where j=0,1,⋯2n, κ(λ)=sech(πλ) is the inverse Fourier transform of the $\chi^{2}$ signature, *n* and *L* are the sampling parameters (they are empirically fixed equal to 3 and 0.65, respectively).

## 5. 3D Model and Architectural Details Localization

Estimating the structure from a set of images depicting a certain scene is a well studied problem in literature [37,38,39,40]. Structure from Motion (SfM) algorithms analyze image matching based on local features in order to simultaneously infer the 3D geometry of the scene and the camera pose for each picture. After finding a set of geometrically consistent matches between each image pair, such matches are organized into tracks. Each track is a set of matching keypoints across multiple images. To recover both the set of camera parameters (*i.e.*, location, orientation, focal length and radial distortion) and a 3D position for each track, the Bundle Adjustment algorithm (BA), which minimizes the reprojection error (the sum of distances between the projections of the track and its corresponding keypoints), is used [39].

To avoid local minima, a common issue of non-linear least squares solvers, it is recommended to perform the optimization incrementally, rather than finding the minimum cost in a single run. Therefore, the minimization is evaluated starting from a pair of images, and then refined adding one image at a time. As the initial pair, we choose images having the largest number of correspondences, given that such matches cannot be modeled by a single homography. This constraint forces a significant change in point of view, therefore a better parameter esteem in the first step. Then, BA keeps adding an image and solving the minimization iteratively. At each step, it selects as a candidate the image having more tracks whose 3D locations have already been estimated, and the optimization is performed again.

As stated, the error function is defined as the sum of the difference between the observed 2D locations and the projections of the corresponding 3D point on the image plane of the camera. More formally, let *p* be the parameter vector (*i.e.*, intrinsic and extrinsic camera parameters, as well as 3D keypoint locations) and f(p) be the reprojection errors for such set of parameters. The optimization can be defined as:

(7) $$\hat{p}=\arg\min_{p}{∥f(p)∥}^{2}$$

The Levenberg–Marquardt algorithm (LM) provides a solution to this problem computing a series of regularized linear approximations to a problem which is non-linear. Calling *J* the Jacobian of f(p), LM solves the following linear problem at each iteration:

(8) $$\left(J^{T}J+\lambda D^{T}D\right)\delta =-J^{T}f$$

where *λ* is an arbitrary regularization term and *D* is a non-negative diagonal matrix. Then, *p* can be updated as:

(9) $$p\leftarrow p+\delta \;\;if\;∥f(p+\delta )∥<∥f(x)∥$$

The matrix $H_{\lambda }=J^{T}J+\lambda D^{T}D$ is known as the augmented Hessian matrix.

To solve this problem with a large photo collection of a cultural heritage building, we propose to use the multi-core bundle adjustment proposed in [41]. This approach shows that inexact step Levenberg–Marquardt can be implemented without storing any Hessian or Jacobian matrices into memory. This allows us to exploit hardware parallelism and to obtain good balance between speed and accuracy.

**Figure 3:**
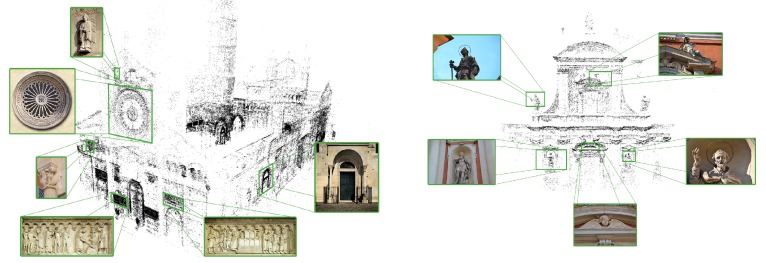
3D reconstruction and relevant details of Cathedral and San Giorgio’s Church in Modena (Italy).

**Figure 4:**
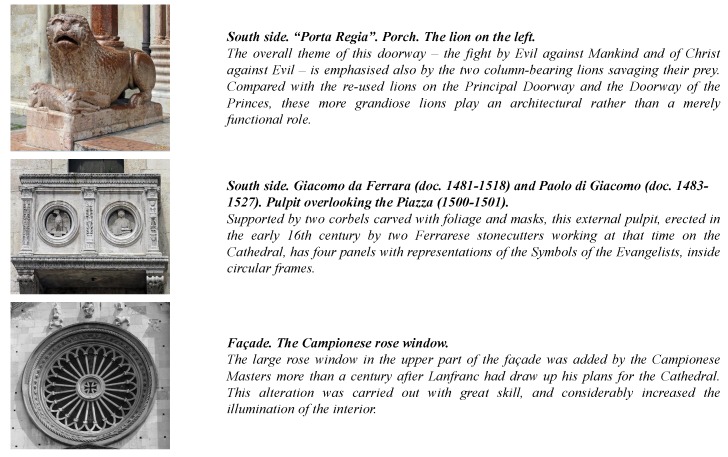
Some exemplar notes stored in the database of the architectural details of the Modena Cathedral.

Once the building 3D model has been estimated, cultural heritage experts have been asked to identify the most interesting architectural details. Figure 3 shows the results of this operation (Cathedral and San Giorgio’s Church, Modena—Italy). Furthermore, a database containing information concerning each architectural detail is built. In particular, each detail has a multi-language description tuned for different user profiles, namely child, casual and expert. Contextual information such as historical notes, artists biographies and multimedia content is also added to the database and accessible through the mobile application (see Figure 4).

**Figure 5:**
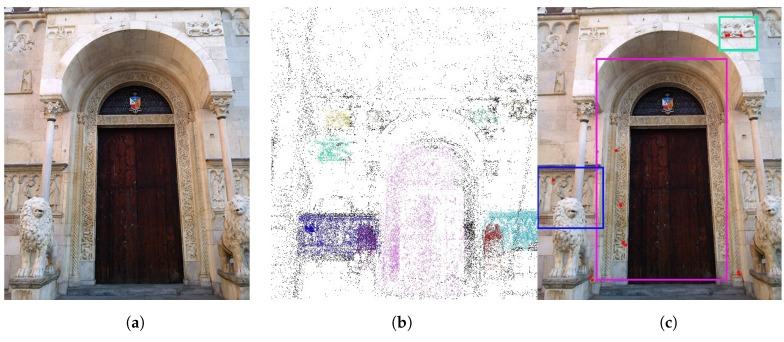
An example of the main steps of our retrieval system: the user query, the retrieval on the point cloud and the reprojection on the original image. (**a**) User input query; (**b**) Retrieved details on 3D model; (**c**) Reprojected points and details.

As output of the procedure, each source image is associated with a set of camera parameters and a 3D position (from where the photo was taken). It is possible to use such information to estimate the absolute pose of a new image and highlight relevant architectural details (see Figure 5).

## 6. Experimental Results

To test our system, we evaluate its performance on two different problems: comparing the proposed image descriptor based on covariance of local features with a large variety of visual descriptors based on BoW and evaluating the user experience in real scenarios. To evaluate the core functionality of the retrieval algorithm, we acquire and publicly release (http://imagelab.unimore.it/files/dataset.zip) a new and challenging dataset that revolves around the romanic cathedral of Modena. It features 743 high quality images capturing different views and different architectural details of the cathedral in different lighting conditions, fully annotated with 10 different possible queries taking into account the whole structure or individual details. The dataset also contains 20 sample query images taken from Google that can be used to reproduce the described results.

### 6.1. Image Ranking

For the first component of our method, we experimentally evaluate is the retrieval algorithm. To show its superior performance, we compare our descriptor to several recent visual descriptors extracted by the implementation proposed by [42]: color moments, generalized color moments up to the second order, giving a 27-dimensional shift-invariant descriptor; RGB Histograms, a combination of three histograms based on the R, G, and B channels; RG Histograms; Hue Histograms; Opponent Histograms; Transformed Color Histograms, RGB histograms obtained by normalizing the pixel value distributions, that achieve scale-invariance and shift-invariance with respect to light intensity; SIFT; RGB-SIFT; RG-SIFT; HSV-SIFT; Hue-SIFT; Opponent-SIFT and C-SIFT, obtained using a C-invariant color space which eliminates the remaining intensity information from the opponent channels.

In all cases, to sparsely sample the keypoints in the images we adopt the *Harris-Laplace* keypoint detector. Since all these are local descriptors, a Bag of Words approach is employed to obtain a global description of the image in order to perform the retrieval phase. Using this approach, a 4096-dimensional codebook is built and used to compute the codes of both dataset images and the query ones. The requirement of a codebook is indeed a liability in this context, since it has two major drawbacks: the first one is the time required to compute it, which, in the worst case, has been of more than 260,000 s (more than three days) on an Intel i7 3.0 GHz CPU. This preprocessing step must be taken for each descriptor type and due to the complexity of the clustering step, which does not depend on the number of images, cannot be shortened without decreasing the precision of the procedure. Our method does not require the computation of a codebook prior to being able to employ the descriptor, effectively saving significant time. On the other hand, being tied to a codebook computed on a particular dataset inevitably leads to being dependent from the training data. Hence, our algorithm is better suited to generalizing than any of the different descriptors it is compared to.

Evaluating our method, we show its performance and we compare it against the aforementioned techniques in terms of Precision@1, Precision@3, Precision@5 and Mean Average Precision (MAP). These metrics respectively show the precision of the first *k*, with k∈{1,3,5}, top ranked images and the overall precision. As the results in Table 1 show, the usage of the covariance of SIFT descriptors leads to the best results. While other SIFT-based approaches can achieve good performance, it clearly emerges how relying only on color information is not sufficient to perform an effective retrieval. This is mainly due to the complexity of the setting we are dealing with, where most of the architectural details share the same color patterns. Indeed this is the reason why most of the descriptors based on color histograms show very poor performances. Computing the gradient information using the SIFT descriptor on different color spaces can achieve slightly better results than relying only on grayscale information. Nonetheless, using our pCov descriptor, which does not rely on color information, can achieve better results than any of the SIFT-based BoW descriptors, whether they use color or not. Further extending the pCov descriptor by applying the explicit feature map described in Section 4 is shown to improve the performance of the method in terms of Precision@k, while slightly degrading the overall MAP. This is still an improvement since our method requires stable results in the first k ranked images and is not affected by noise in the lower parts of the ranked list.

### 6.2. Wearable Devices Performance

To improve the user experience by providing a smoother application capable of lower response times, we adopt the new wearable platform, the Jetson TK1 board, with respect to the one used in [11]. Featuring a Tegra K1 GPU with 192 CUDA cores, this device allows to write GPU-parallelized code combining the lightweight of a wearable system with the computational advantages of modern GPU programming. To understand the potential advantages of parallelizing on a GPU our algorithm, we evaluate the computational demands of its different components. In fact, its main steps are the SIFT feature extraction on a newly acquired frame, the computation of its pCov descriptor, the extraction of the explicit feature map using the Homogeneous Kernel Map and the matching of the frame descriptor with the image database (see Section 2 for more details).

**Figure 6:**
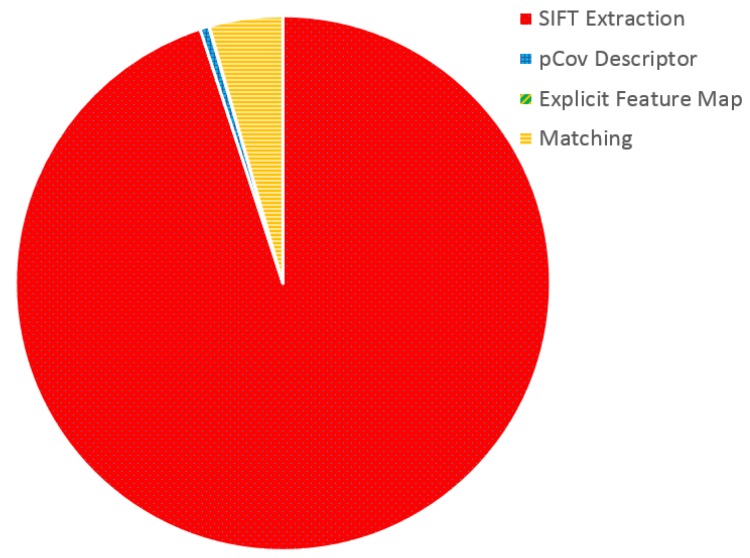
Evaluation of the computational demands of the different steps of our algorithm. Note that the time required by the Explicit Feature Map is negligible and cannot be seen in the chart due to the scale of the plot.

**Figure 7:**
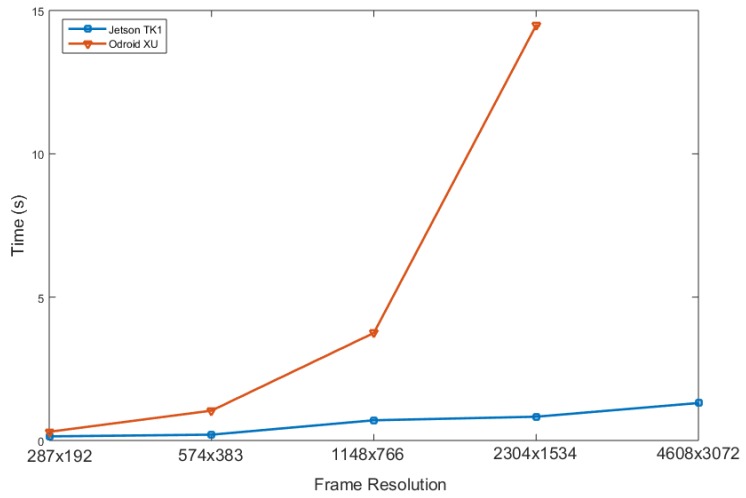
Comparison between Jetson TK1 and Odroid XU wearable boards on the task of SIFT extraction.

Figure 6 reports the results of such evaluation. It can be noticed how nearly 95% of the execution time is spent in SIFT descriptor extraction. Following this result, we parallelize the extraction of SIFT features from the newly acquired frame and run it on the device GPU, obtaining a significant improvement. To measure the impact of this process, we evaluate the execution time required by the SIFT extraction process on two different wearable devices, the Nvidia Jetson TK1 (with GPU parallelization) and the Odroid XU, the wearable device adopted in [11]. Figure 7 shows the results of this comparison. In particular, to better capture the contributions of exploiting the Jetson TK1 GPU, the two architectures are evaluated under different image resolutions. It can be noticed that the Odroid XU board suffers from an exponential increase in time required to compute the SIFT descriptors when increasing the frame resolution by a factor of two. Furthermore, due to the limited hardware resources of the board, it cannot process images of a resolution of 4608×3072 reducing the potential scalability of the system. On the other hand, the Jetson TK1 board features significantly lower execution times, going down from the 1.041 s of the Odroid board to 0.203 in the case of a resolution of 574×383.

To provide a quantitative evaluation of the impact of different resolutions of the extraction of SIFT descriptors, we report in Table 2 the number of features found by the algorithm at different resolutions. These results show that a resolution of 574×383 produces a sufficiently high amount of features in only 200 ms, effectively allowing for a nearly real-time response to the user after a query. Higher resolutions could be used and, for example, in the case of 1148×766 the adoption of the Jetson TK1 board still provides a speedup of more than five times with respect to the Odroid XU board.

### 6.3. User Evaluation

This evaluation should aim at establishing how the users respond to this new kind of technology, in terms of how they enjoy it, how natural does the interaction feel and how effective the tourists deem the application to be. With these objectives in mind, we staged an evaluation that involved 40 people of different sex and ages (20–40 years old). To provide a measure of the background knowledge of technology of the people who volunteered for the study, we provide information about the education background of the participants. In particular, the study involved eight university students (humanistic studies), 26 university students (engineering), and six Ph.D. students (engineering). Each one of them has been provided with a prototype of our system and has been accompanied in a small tour of Modena, focusing on the cathedral. Here, the tourist took the chance to test our system and, after the visit, had to respond to a brief questionnaire. We asked them to respond to a few questions using a Likert scale with scores from 1 (lower) to 5 (higher). The two questions were: “Express how much did you enjoy using the tool” and “Describe how effective it was”, where the effectiveness was expressed in terms of how many architectural details you would otherwise have missed.

**Figure 8:**
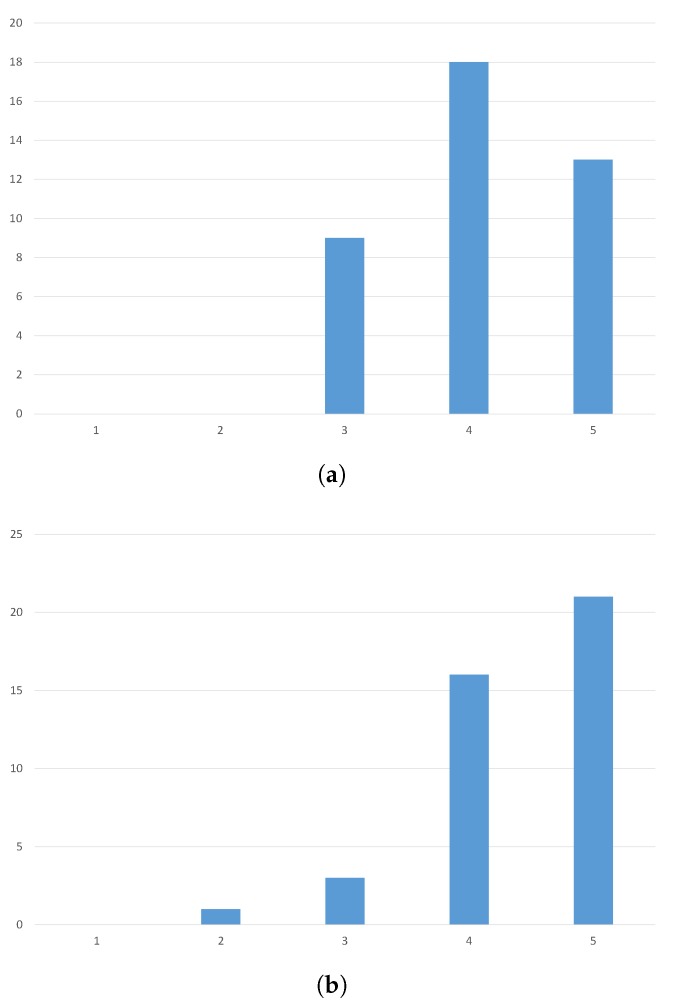
The results of the user experience evaluation of the proposed system. (**a**) “Express how much did you enjoy using the tool”; (**b**) “Describe how effective it was”.

The results of this interrogation, that can be seen in Figure 8, validate the proposed system. In fact, 80% of the users evaluated the system as enjoyable (score of 4 or more). When asked to describe how useful the tool is in terms of how helpful it can be in localizing the details on the building, 90% of the users positively responded. These results indicate that this technology will be able to effectively improve cultural experiences.

## 7. Conclusions

In this paper, we proposed a method to enrich the user experience when visiting a cultural heritage site. By exploiting the new egocentric paradigm, a tourist can explore a historical landmark and retrieve its architectural details, and ask for information such as historical background or authors’ biographies either via audio or a mobile application. To achieve this goal, we proposed a novel image descriptor based on the covariance of local SIFT features that can achieve state-of-the-art accuracy and can be efficiently computed on a wearable device. Exploiting the recent advancements in GPU programming, we speed up the most onerous component of the method and achieve real-time performance, a critical factor in an application that aims at improving a user experience. A 3D model of a cultural heritage building is also computed and annotated with relevant architectural detail by cultural heritage experts. Given a query acquired by the visitor, using this image retrieval solution, we can achieve a stable ranking of the most similar images relating to the 3D point cloud, obtaining an accurate user localization and a precise detail identification. This results in an effective enhancement of the cultural experience, as confirmed by the user evaluation performed on the field. As future work, we plan to further extend the user evaluation through a more complete set of questionnaires based on ISO standards to better analyze the reliability and usability of the system.

## Figures and Tables

**Table 1:** Comparison between different descriptors employed in our evaluation. pCov-SIFT is the projected covariance descriptor (see Section 4) and pCov-SIFT + EFM (Explicit Feature Map) is the projected covariance descriptor using the explicit feature map (see Section 4).

Descriptor	Precision@1	Precision@3	Precision@5	MAP
pCov-SIFT + EFM	0.850	0.750	0.650	0.339
pCov-SIFT	0.800	0.717	0.600	0.362
RGB SIFT	0.750	0.650	0.570	0.281
Opponent SIFT	0.750	0.600	0.500	0.266
SIFT	0.750	0.617	0.550	0.268
RG SIFT	0.650	0.483	0.420	0.235
C-SIFT	0.600	0.567	0.430	0.236
HSV-SIFT	0.560	0.483	0.440	0.203
Transformed Color Histograms	0.500	0.283	0.300	0.187
Hue SIFT	0.450	0.350	0.260	0.129
Opponent Histograms	0.300	0.233	0.210	0.109
RGB Histograms	0.300	0.217	0.190	0.112
Color Moments	0.200	0.150	0.170	0.115
RG Histograms	0.150	0.133	0.140	0.098
Hue Histograms	0.100	0.100	0.100	0.088

**Table 2:** Average number of SIFT features extracted at different frame resolutions.

Resolution	# Features
287×192	529
574×383	2166
1148×766	8847
2304×1534	10307
4608×3072	15732
